# Temporal Regularity of the Environment Drives Time Perception

**DOI:** 10.1371/journal.pone.0159842

**Published:** 2016-07-21

**Authors:** Darren Rhodes, Massimiliano Di Luca

**Affiliations:** 1 Centre for Computational Neuroscience & Cognitive Robotics, School of Psychology, University of Birmingham, Edgbaston, Birmingham, United Kingdom; 2 Sackler Centre for Consciousness Science, School of Engineering & Informatics, University of Sussex, Brighton, United Kingdom; University of Groningen, NETHERLANDS

## Abstract

It’s reasonable to assume that a regularly paced sequence should be perceived as regular, but here we show that perceived regularity depends on the context in which the sequence is embedded. We presented one group of participants with perceptually regularly paced sequences, and another group of participants with mostly irregularly paced sequences (75% irregular, 25% regular). The timing of the final stimulus in each sequence could be varied. In one experiment, we asked whether the last stimulus was regular or not. We found that participants exposed to an irregular environment frequently reported perfectly regularly paced stimuli to be irregular. In a second experiment, we asked participants to judge whether the final stimulus was presented before or after a flash. In this way, we were able to determine distortions in temporal perception as changes in the timing necessary for the sound and the flash to be perceived synchronous. We found that within a regular context, the perceived timing of deviant last stimuli changed so that the relative anisochrony appeared to be perceptually decreased. In the irregular context, the perceived timing of irregular stimuli following a regular sequence was not affected. These observations suggest that humans use temporal expectations to evaluate the regularity of sequences and that expectations are combined with sensory stimuli to adapt perceived timing to follow the statistics of the environment. Expectations can be seen as a-priori probabilities on which perceived timing of stimuli depend.

## Introduction

Time is a physical dimension that pervades numerous aspects of human perception, yet humans do not perceive time veridically. For example, the subjective experience of duration can be modulated by non-temporal characteristics such as stimulus properties [1–3], complexity [4], sensory modality [5–7], and context [8]. As subjective duration can be biased by the characteristics of the immediate environment, here we ask whether the perception of temporal regularity is subject to similar influences. In fact, we observe that context plays a role in the perception of rhythmic stimuli, as humans effortlessly learn the temporal structure of events [9–14], an ability that is present even in newborns and infants [15,16]. It has been shown that humans, among other animals [17–22], can improve perceptual judgments [23–29], reduce neural metabolic consumption [30], and automatize behaviour [31,32] by entraining to regular rhythms. Whilst there are several models of how humans deal with rhythmic stimuli [33–44], these accounts assume that if stimuli are repeated after the same inter-stimulus interval, then they are perceived as part of a regular sequence. But, as previously noted, time is subject to perceptual distortions, so why should a temporal property like regularity be immune to these too?

To test whether perceived regularity of a sequence can change due to the influence of the environment, we presented sequences of stimuli that were perceptually regularly timed to one group of participants, whereas a second group was presented with mostly irregularly timed sequences. In both groups, 25% of trials comprised a regular sequence, i.e., stimuli spaced by the same inter-onset interval (isochrony). We found that an environment of mostly irregularly timed stimuli causes regular sequences to appear as being irregular. We don’t find support for explanations based on response biases or changes in the criterion for judging regularity, and thus we propose an explanation of these effects that is based on the influence of expectations on the perceived timing of individual stimuli; i.e., humans make predictions about the timing of future events that modify perception. An ideal candidate to conceptualise this phenomenon is *Bayesian Decision Theory* (BDT), which has been successfully applied to various perceptual domains [45–49]. Bayesian accounts of time perception have also been formulated [44,50–54], but they do not account for changes in the perceived timing of individual stimuli as they are contingent on the representation of duration that stimuli delimit. In contrast, we hypothesized that humans represent the timing of events separately from duration for the purpose of estimating temporal properties of the stimulus sequence, i.e., regularity. Such a scheme relies on previous knowledge about the timing of stimuli. In a regular environment, perceived timing of stimuli is obtained by the combination of incoming sensory information with expectations of when such stimuli should appear. Expected timing should thus bias perception in a way that regularises slightly anisochronous stimuli. To test this, we measured when stimuli at the end of a sequence are perceived and found a temporal regularisation effect, such that the timing difference between early and late stimuli is perceptually reduced. Our results give more credence to the contemporary idea [50,52,53,55,56] that the brain keeps track of the temporal statistics of the environment and uses them for perception in a way consistent with Bayesian inference.

## Experiment 1: The Temporal Environment Modifies Perceived Regularity

**Fig 1:**
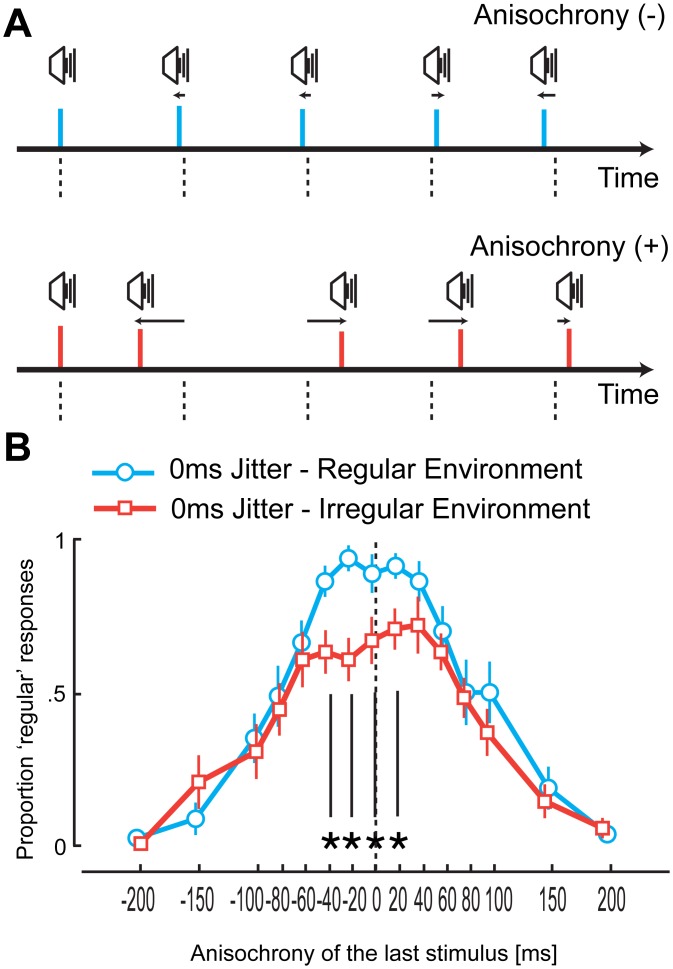
Experimental stimuli, design, and results of Experiment 1. (A) Top: Example of a sequence in a perceptually regular environment with the final stimulus earlier than expected. Bottom: Example of a sequence in a perceptually irregular environment with the final stimulus later than expected. (B) Proportion of ‘regular’ responses as a function of the timing of the final auditory stimulus in the sequence. Each line represents data obtained with 0 ms jitter level for the two groups. When comparing the data obtained from the identical 0 ms jitter level sequences presented in both groups, the number of ‘regular’ responses is significantly lower for the perceptually irregular group at the points denoted by an asterisk (S2 Table). In all graphs, the error bars represent the standard error of the mean.

The first experiment examined whether the regularity of the environment changes how temporal regularity of stimulus sequences is perceived. We exposed participants either to an environment where the majority of sequences were perceived to be temporally regular (regular environment, Fig 1A upper panel) or to an environment where most of the sequences appeared to be markedly irregular (irregular environment, Fig 1A lower panel). To assess participants’ sensitivity to discriminate whether a sequence appears regular [57–59], for both groups we presented 25% of sequences composed of five beeps where only the timing of the last stimulus deviated from isochrony. The participants’ task was to report whether the sequence as a whole was regular–or not. By parametrically varying the timing of the last stimulus we determined the deviations at which sequences started to appear irregular.

### Method

#### Participants

Twenty undergraduate students from the University of Birmingham took part in this experiment. In both of the experiments presented in this article, written informed consent was given in accordance with the Declaration of Helsinki. The Science, Technology, Engineering & Mathematics Ethical Review Committee of the University of Birmingham approved both experiments. Participants were recruited via the online participant recruitment system and received course credits as reimbursement. Participants were naïve to the purpose of the study, and all had normal or corrected-to-normal hearing or vision.

#### Stimuli

The auditory stimuli were identical tones produced by a speaker positioned 50 cm to the left of the participant (20 ms with 5 ms linear ramp, 1 kHz, 75.1 dBA). To ensure reliable timing, the signals of the whole trial were sent to the audio card before presentation.

#### Procedure

Participants sat in a quiet, well-lit room, 50 cm away from a speaker positioned to their left. The design of the experiment included a between-subjects factor such that 10 participants partook in the ‘regular’ environment, whilst 10 participants took part in the ‘irregular’ environment. After being given instructions and informed consent, participants were presented with sequences of five auditory beeps with an inter-onset interval (IOI) of 700 ms that could be highly irregular (irregular environment group: 0, 50, 100, or 150 ms jitter level) or perceptually regular (regular environment group: 0, 10, 20, or 30 ms jitter level). The jitter was applied to the regular sequence, such that a uniformly distributed random value sampled between +/− the jitter level was added to each IOI. (The procedure is identical in Experiment 2.) Conditions were randomly interleaved such that the participant could not know the jitter level of the next stimulus sequence. The participants’ two-alternative forced choice task (2AFC) was to report whether the stimulus sequence was regular or not by pressing one of two keys (Fig 1). The final stimulus could be temporally deviant by 0, ±20, ±40, ±60, ±80, ±100, ±150, or ±200 ms. Participants were asked to respond as accurately as possible with no time limit on the response window. After pressing the regular or irregular key, the next trial would start after 1.5–2 seconds. Each condition was presented eight times. The experiment lasted approximately 30 minutes, and participants were debriefed upon completion of the task.

### Results

**Fig 2:**
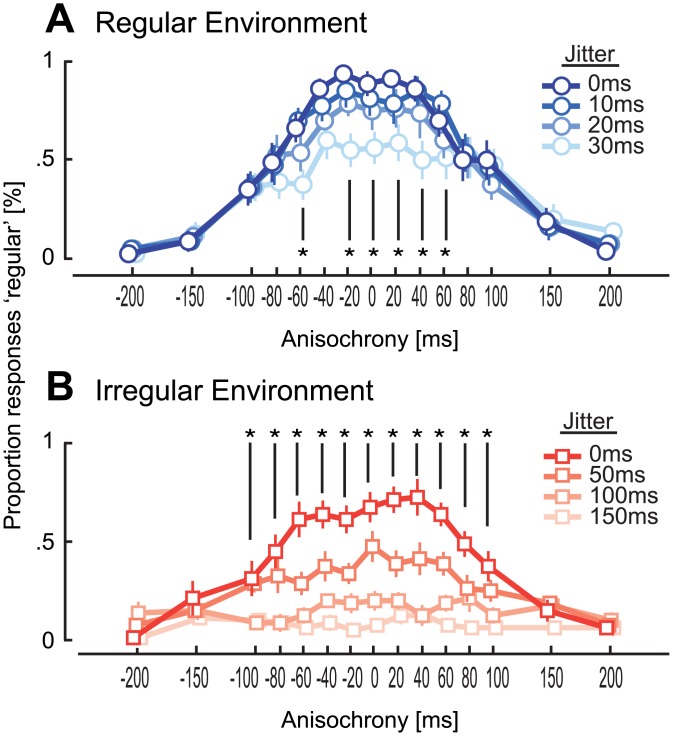
Additional analysis of Experiment 1. Proportion of ‘regular’ responses as a function of the timing of the final stimulus in the sequence (A) for the perceptually regular environment group and (B) for the perceptually irregular environment group. Each line represents one of the four jitter levels. The distribution of responses becomes steeper at lower jitter levels for each group. Asterisks denote anisochronies at which the proportion of responses differs significantly across jitters levels within a group (see S1 Table for statistical tests).

**Fig 3:**
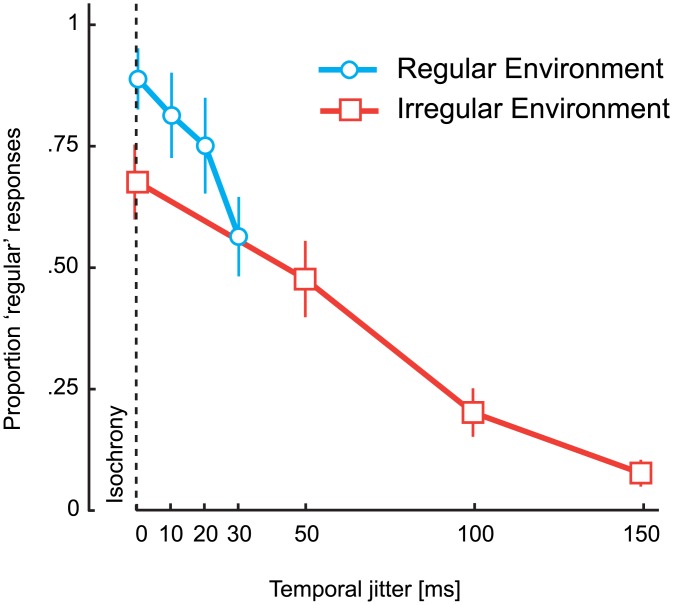
Proportion of regular responses as a function of the jitter level for 0 ms anisochrony of the final stimulus. Participants respond more frequently ‘regular’ when the final stimulus follows a sequence of perfectly regularly timed stimuli in the regular environment (blue line) in comparison to an irregular environment (red line).

The distribution of responses as a function of jitter level became steeper in both environments (Fig 2) as shown by the significant interaction term in a two-way repeated measures ANOVA with factors anisochrony of final stimulus and jitter level on the inverse-normal proportion of ‘regular’ responses bounded between .01–.99 (regular: *F*(42,378) = 3.5, *p* < .0001, η_p_^2^ = .28, irregular: *F*(42,378) = 5.0, *p* < .0001, *η*_*p*_^*2*^ = .36). On the other hand, the distribution of responses for sequences composed of four initial regularly timed stimuli (0 jitter level) as a function of the deviation of the last stimulus followed a different pattern between the two groups (Figs 1B and 2, see S1 Table for responses with jittered sequences in both groups). A mixed ANOVA on the proportion of regular responses in 0 jitter level sequences with a within subject factor of anisochrony of final stimulus and between groups factor of environment (regular/irregular) revealed an expected significant main effect of anisochrony (*F*(14,252) = 3.4, *p* < .001, η_p_^2^ = .16), but no effect of environment (*F*(1,18) = 0.3, *p* = .572, η_p_^2^ = .02) and, more importantly, a significant interaction (*F*(14,252) = 36.2, *p* < .0001, η_p_^2^ = .67). For participants exposed to an irregular environment, the number of ‘regular’ responses for slight deviations in the timing of the last stimulus appeared to be much lower than the same sequence embedded in the regular environment (significant between -40 and 20 ms, see S2 Table for independent-sample *t*-tests between each environment). Crucially, participants were also more likely to report perfectly regular sequences (0 ms anisochrony) as being irregular when embedded in an irregular environment. The difference between the two groups disappears for larger deviations of the last stimulus in the sequence. Furthermore, when the last stimulus had 0 ms anisochrony, participants perceive regularity the most when the first part of the sequence is isochronous and the proportion of responses declines almost linearly as a function of the jitter level (Fig 3).

To test whether a change in the decisional criterion used to give ‘regular’ answers could be an explanation for the pattern of results, we applied the two noisy criteria simultaneity judgment model [60], hypothesizing that participants respond ‘regular’ if the sensed value of anisochrony is contained within two criteria, one for each of the directions of anisochrony. According to the original formulation, the location of the two criteria is drawn from two Gaussian distributions with independent variability, which may cause the slope at the two sides of a psychometric function to be different. The four parameters of the model are the anisochrony of the early (negative anisochronies) and late (positive anisochronies) criteria for judging regularity and the slopes at the threshold points. Using the tool described in [60], we analysed the data of the initially regularly timed sequences (0 jitter level) by fitting the proportion of responses as a function of anisochrony using the difference of two cumulative Gaussian distributions. The values for all the parameters are presented in S3 Table. The fit did not evidence changes between regular and irregular environment groups for any of the parameters (paired sample t-tests and Bayes Factors on thresholds for early *t*(18) = 0.2, *p* = .82, *BF*_*10*_ = .41; late *t*(18) = -0.7, *p* = .51, *BF*_*10*_ = .47; on slope for early *t*(18) = -1.7, *p* = .20, *BF*_*10*_ = 1.0; late *t*(18) = -2.3, *p* = .060, *BF*_*10*_ = 2.3). By ruling out that a change in the response criterion, we increase our confidence in concluding that the environment had a genuine influence on the perception of sequence regularity.

## Experiment 2: The Temporal Environment Changes Perceived Timing

**Fig 4:**
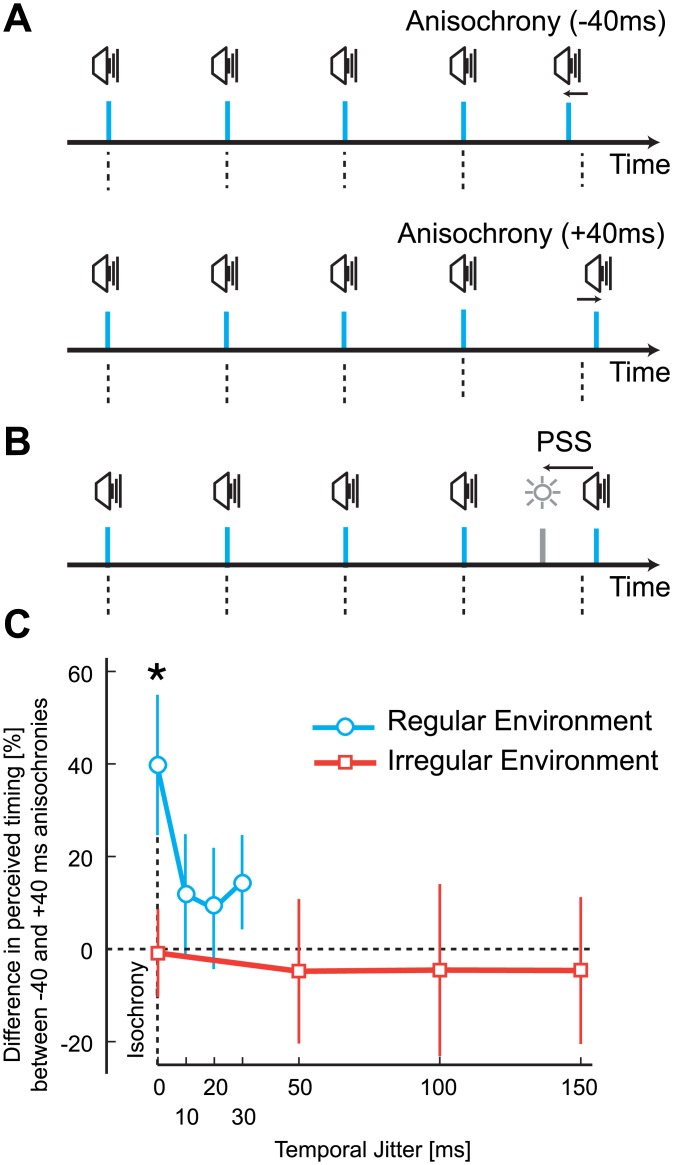
Experimental stimuli, design, and results of Experiment 2. (A) Examples of trial sequences in regular environments when the final stimulus was earlier (top panel) or later (bottom panel) than expected. Participants judged the temporal order of the final auditory stimulus and a visual probe (B), which was used to determine the point of subjective simultaneity (PSS). (C) Difference in perceived timing (as a percentage) between stimuli presented -40 ms earlier than expected and +40 ms later than expected as a function of the jitter level. Each line represents the two environment groups.

Here we posit that since perceived regularity of isochronous sequences may decrease if sequences are embedded in a temporally irregular environment, the opposite could be also true–the brain could have a mechanism that makes slightly anisochronous sequences appear more regular if embedded in a temporally regular environment. This could be achieved if the perceived timing of individual stimuli is modified so that they appear closer in time to the regular time point. To test whether the perceived timing of a temporally deviant stimulus is modified, we asked participants to report whether the final stimulus in an auditory sequence was presented before or after a temporal probe in the visual modality (Fig 4A and 4B). To measure the perceived timing of the last stimulus, we compared the physical asynchrony at which audio and visual stimuli are perceived to be simultaneous (PSS, Point of Subjective Simultaneity) for stimuli presented slightly earlier and later than isochrony. If the brain regularises these stimuli, we should find that the perceived timing modifies in a way consistent with a delay if the stimulus is presented too early and/or with an acceleration of the stimulus if it is presented too late.

### Method

#### Participants

Twenty-four undergraduate students were recruited via the University of Birmingham research participation system and received course credits for their participation. All participants reported normal or corrected-to-normal hearing and vision, and all were naïve to the purpose of the study.

#### Stimuli

The auditory stimuli were five identical tones produced by a speaker approximately 50 cm to the left of the participant (20 ms with 5 ms linear ramp, 1 kHz, 75.1 dBA). Visual stimuli were red flashes of light produced by a 5 mm LED (20 ms with 5 ms linear ramp, 91 Cd/m^2^) positioned directly in front of the participant and 20 cm to the right of the auditory stimuli. The signals were generated on MATLAB using the psychophysics toolbox extension [61,62] and sent to the audio card before presentation to ensure reliable timing.

#### Procedure

Participants sat approximately 50 cm from the custom-made light and sound devices in a well-lit, quiet room. The design of the experiment included a between-subjects factor. Ten participants partook in the ‘irregular’ environment and 10 participants took part in the ‘regular’ environment. Participants performed the experiment in two phases. In the *practice phase* participants performed a 2AFC temporal-order judgment task (“did the light or the sound come first?”) with stimuli separated by an SOA of 0, ± 20, ± 90, ± 170, ± 250, or ± 350 ms. Each condition was repeated six times. The next trial would begin 1.5 or 2 s after the response key had been pressed. The purpose of the practice phase was to identify whether the participant could successfully discriminate temporal order.

In the *test phase*, one group of participants was exposed to sequences of stimuli that were perceptually isochronous (jitter level = 0, 10, 20, or 30 ms) whilst a second group was exposed to perceptually anisochronous sequences (jitter level = 0, 50, 100, or 150 ms). In each trial, four auditory stimuli were presented with an IOI of 700 ms. For both groups, 25% of the trials comprised perfectly isochronous sequences (0 jitter level). The participant’s task was to decide whether the last auditory stimulus in the sequence was before or after a visual probe stimulus (2AFC: did the sound or light come first?). The audio and visual stimuli were presented with one of nine SOAs (0, ±40, ±80, ±120, or ±200 ms). Critically, the last auditory stimulus in the sequence was presented ± 40 ms earlier or later than expected (Fig 4A; negative values mean the stimulus was presented earlier than expected). Each condition was repeated 10 times.

#### Psychophysical Analysis

To detect changes in the perceived timing of stimuli, the point of subjective simultaneity (PSS) and just noticeable difference (JND) were obtained from the proportion of responses as a function of SOA. We define the PSS as the SOA at which each subject was equally likely to report that the visual probe or last repeated stimulus was first. Negative PSS values mean that sound had to be presented before light in order to be perceived as synchronous, whilst positive values mean that light had to be presented before sound to be perceived as synchronous (Fig 4B). We define the JND as the SOA that needs to be added or subtracted to the PSS to obtain .16 or .84 proportion of responses. We used the Spearman-Kärber method to obtain the PSS and JND as the first and second moments of the differential of the distribution of responses (see [63] for further description of method). The method is non-parametric, as it does not make assumptions about the shape of the distribution that underlies the psychometric function. A mathematical derivation of the method can be expressed as follows: First, we define SOA_i_ with i = {1,… 15} as the 15 values of SOA used in the experiments and p_i_ with i = {1, … 15} as the associated proportion of ‘light first’ responses. We further define SOA_0_ = -250 ms, SOA_16_ = +250 ms, p_0_ = 0, and p_16_ = 1, so that we can compute the intermediate SOA between two successive ones

$${\text{s}}_{\text{i}}=\;\frac{{\text{SOA}}_{\text{i}+1}+{\text{SOA}}_{\text{i}}}{2},\text{ with i}=\left\{0,\;\ldots \;15\right\}$$ (1)

and the associated values of the difference in proportion

$${\text{dp}}_{\text{i}}={\text{p}}_{\text{i}+1}-{\text{ p}}_{\text{i}},\text{ with i}=\left\{0,\;\ldots \;15\right\}.$$ (2)

With these indexes we can express PSS analytically as:

$$\text{PSS}=\;\frac{1}{\sum_{\text{i}=0}^{15}{\text{dp}}_{\text{i}}}\;\sum_{\text{i}=0}^{15}{\text{s}}_{\text{i}}{\text{dp}}_{\text{i}}$$ (3)

and the JND as:

$$\text{JND}=\;\sqrt{\sum_{\text{i}=0}^{15}{\text{dp}}_{\text{i}}{\left({\text{s}}_{\text{i}}-\text{PSS}\right)}^{2}}.$$ (4)

We intended not to analyse participant’s data if JND was above 250 ms in the practice phase or 200 ms in the test phase, however all participants’ performance exceeded this criterion.

### Results

**Fig 5:**
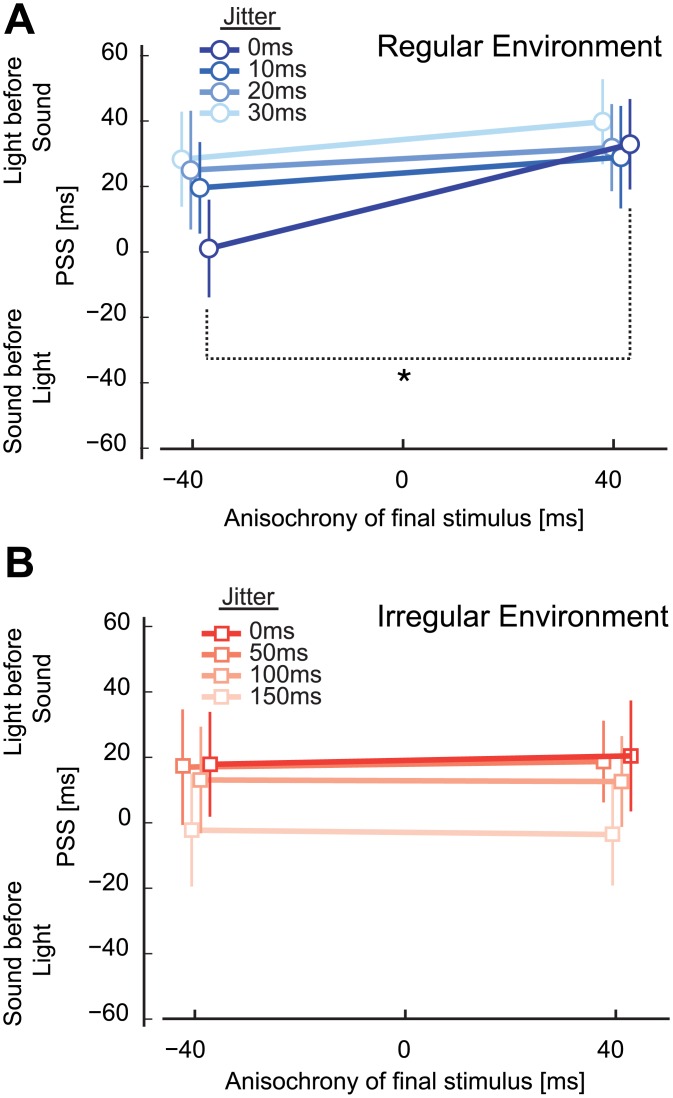
PSS values of Experiment 2 decomposed into early and late anisochrony. Average PSS values corresponding to the SOA at which both the audio and visual stimulus were perceived as simultaneous. Positive PSS values indicate that the light needs to be presented before the sound to be perceived as simultaneous whilst negative values indicate that the sound has to be presented before the light to be perceived as simultaneous. PSS values should be horizontal and not change if the stimulus anisochrony does not affect the perceived timing, instead the PSS values appear to change for 0 ms jitter level in the (A) regular environment group, whilst no such change is exhibited in the (B) irregular environment group.

For sequences in the 0 jitter level condition (the four initial stimuli were isochronous) we found a change in PSS between early and late stimuli only for the group exposed to a regular environment. A mixed two-way ANOVA on PSS values with within-subject factor anisochrony (-40/40 ms) and between-subject factor group (regular/irregular environment) revealed a main effect of anisochrony, *F*(1,22) = 6.1, *p* = .022, η_p_^2^ = .22, and the critical interaction *F*(1,22) = 4.4, *p* = .047, η_p_^2^ = .17. Subsequently, a t-test between early and late stimuli was significant in the regular environment, *t*(11) = -2.6, *p* = .024, but not in the irregular condition, *t*(11) = -.384, *p* = .078 (Figs 4 and 5). Furthermore, Bayesian t-tests [64,65] support the claim that a difference in PSS between early and late stimuli exists in a regular environment (*BF*_*10*_ = 2.9), with likely no difference in the irregular group (*BF*_*10*_ = .3). Contrasts of PSS difference between environments at each jitter level were not significant for any of the other comparisons. These data suggest that the relative timing between the final auditory stimulus and a visual probe changes in ways consistent with stimuli presented early being processed slower than stimuli presented late. An important assumption here is that changes in the PSS reflect variations in the processing of the repeated auditory stimulus, and not the visual probe [66].

**Fig 6:**
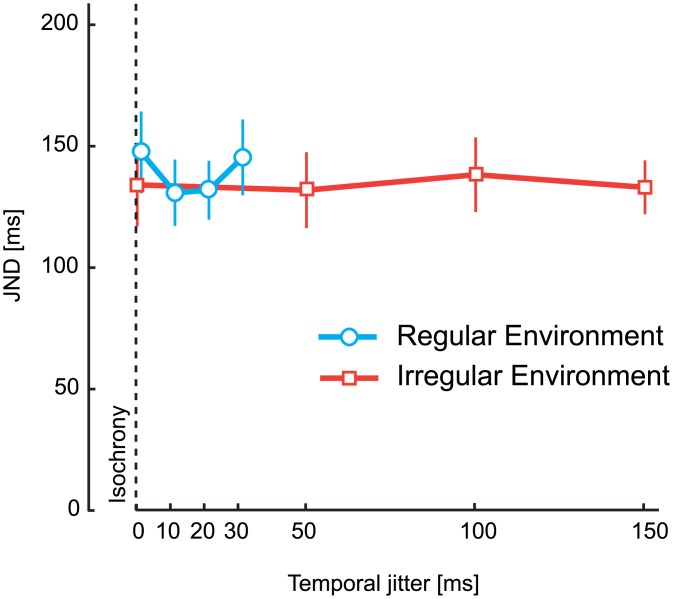
JND values of Experiment 2. JND values for the two environment groups as a function of jitter level. The JND represents how well participants can discriminate between the audio and visual stimulus. A change in JND would suggest that the jitter level affected participants’ ability to discriminate perceived stimulus timing.

The magnitude of the effect (difference in perceived timing between early and late stimuli) was highest for stimuli at the end of an isochronous sequence of four stimuli (40% of the anisochrony +/-15.3% SEM), but the effect was in the same direction for irregular sequences (10, 20, and 30 ms jitter level of the first four stimuli) in the regular environment (Fig 4C). Thus, by comparing early and late stimuli, we found a perceptual reduction in asynchrony that is significantly different from 0 only in the regular group (*t*(11) = 2.6, *p* = .024, *BF*_*10*_ = 2.9), but not in the irregular one (*t*(11) = 0.4, *p* = .708, *BF*_*10*_ = .3), whilst the groups differed from each other, *t*(22) = 2.1, *p* = .047, *BF*_*10*_ = 1.7. No magnitude difference in PSS was registered for the group exposed to an *irregular* environment (Figs 5B and 4C). In other words, participants exposed to a regular environment reported the timing of slightly deviant stimuli at the end of the sequence so that the sequence appears more regular than it actually is–a temporal regularisation. Notably, sensitivity in judging the timing of the audio and visual stimuli did not differ in the two temporal environments. An independent sample t-test on JND values with a between-subjects factor of group (regular/irregular) for the condition with 0 ms jitter level yielded was not significant *t*(22) = 0.4, *p* = .671, *BF*_*10*_ = 0.4 (Fig 6).

## Discussion

In this study, we demonstrate that the degree of temporal regularity in the environment drives perceived stimulus timing. A temporally regular environment leads to sequences being reported to appear regular more often than when the same sequences are presented in an irregular environment. Such a difference is present also for perfectly regular sequences. In principle, this result could be explained in two ways: (a) A response bias that leads participants to respond ‘irregular’ more often in the irregular environment. Such a pattern could be accounted for by a change in the decisional criterion for perceiving sequences to be regular in the irregular environment which tolerates perceptual anisochronies caused by neural noise; or (b) There could be an influence of the regular environment on the perception of the timing of individual stimuli, so that they are perceptually moved towards the expected time points–by delaying stimuli presented earlier than regular and/or by accelerating stimuli presented later than regular.

Our data is not consistent with a response bias effect (a), as here one would expect to see a general increase in ‘irregular’ responses in the irregular context over all levels of anisochrony. However, as Fig 1B illustrates, only with a jitter level of 0 ms is there an increase in ‘irregular’ responses for slightly irregular sequences, suggesting that participants did not change their responses to suit the environment they were exposed to. Moreover, we show that the data of Experiment 1 does not provide evidence suggesting that there are changes in the criterion at which regularity is judged, as we found no difference between groups in the anisochrony of the criteria nor in the sensitivity of regularity discrimination in the 0 jitter level condition [60]. In Experiment 1, we apply this model to regularity judgments as a function of anisochrony. We find no difference between the high (positive anisochronies) and low (negative anisochronies) criteria for judging regularity between regularly and irregularly timed environments. The lack of a difference between groups suggests no differential sensory noise between regular and irregular environments nor differences in the decision uncertainty for judging regularity.

Our results point instead to an explanation based on the change in the perceived timing of stimuli in a sequence due to the presence of a regular environment–irregular stimuli in a regular environment are perceived closer in time to the expected time point (Figs 1 and 2). This effect can be understood as the brain attempting to estimate time from imprecise sensory information as detailed below.

### Bayesian Time Perception

**Fig 7:**
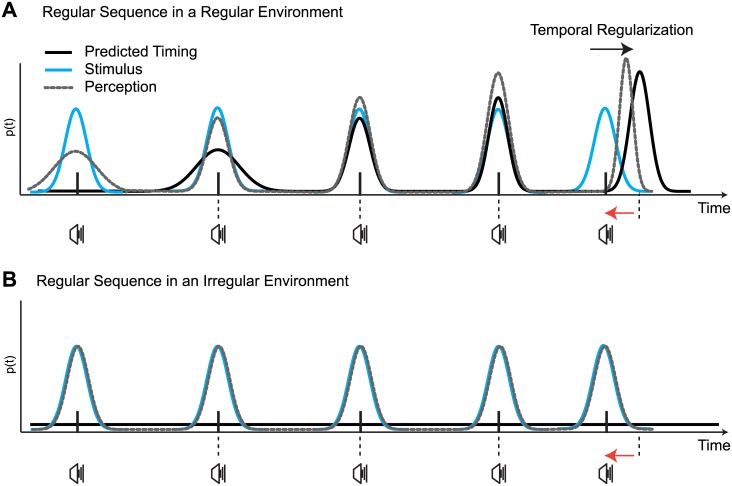
A Bayesian model of perceived regularity. (A) A regular sequence in a regular environment builds up temporal expectations (prior predicted timing; black line) after each presentation of a stimulus (likelihood; blue line) in order to form an estimate of perceived timing from the posterior (perception; grey dotted line). In this way, the posterior becomes the predicted timing for the next stimulus in the sequence. If a final stimulus is presented slightly earlier than expected, then it should be perceptually delayed as well as having greater temporal precision. (B) In a regular sequence in an irregular environment, on the other hand, the posterior distribution coincides with the likelihood, as a flat prior does not contribute to improve precision or generate greater temporal expectations. Lower precision results in a wider posterior distribution and, as such, after each stimulus presentation, a physically regular stimulus could by chance be reported as being irregular.

We interpret our results using a Bayesian framework, considering that the brain is trying to obtain an estimate of the timing for each stimulus from noisy sensory information. To improve the reliability of the estimates, the brain incorporates *a-priori* knowledge (prior probability) of when a future stimulus *may* occur with incoming sensory evidence (likelihood function) of when a stimulus *actually* occurred in the world (Fig 7). We consider that the brain dynamically updates the prior probability of experiencing a stimulus after each presentation. Temporal expectations rapidly increase and bias the timing of stimuli in a regular environment so to make them appear more regular (Fig 7A). In a temporally irregular environment, however, temporal statistics are less precise and thus expected timing is more uncertain (Fig 7B). An estimate of perceived timing is based on the posterior distribution, which is the combination of the prior and likelihood. In an irregular environment, the posterior distribution coincides with the likelihood distribution (as there is no regularity in the sequences, the prior does not contribute to improve precision). Lower precision means a wider distribution, and thus at each encounter of a stimulus, even if physically regular, there is a possibility that the stimulus is actually anisochronous. For stimuli embedded in a regular environment instead, expectations improve the precision of timing estimates, making stimuli appear closer to the presented time point–but they also influence the timing of slightly deviant stimuli. Such an effect is evidenced by asking participants to judge whether the final stimulus in a sequence appears before or after a visual probe (Experiment 2). In a regularly timed environment, slightly anisochronous stimuli are biased to appear closer in time to the expected time point due to the effect of the prior (Fig 4C).

We reason that temporal regularisation reflects the tendency to obtain a compact representation of the environment that can be generally applied for perception [67]. In a regular environment, small temporal irregularities are likely due to sensory noise, and thus representing each individual irregularity would constitute a disadvantageous memory burden. Modifying the perceived timing of stimuli, so to appear more regular, reduces neural metabolic consumption [30] and improves the perceptual processing of regularly timed stimuli [23–29,50].

Current models of temporal perception [13,33,35,38,57,68] are mainly concerned with a representation of the duration of an interval and do not make explicit predictions about whether the temporal environment should influence the perceived timing of temporally deviant stimuli. In fact, in these models, the perceived timing of a stimulus is mostly ignored, because they assume that stimuli are simply perceived after a constant perceptual delay. More importantly, no model relates the change in detection of temporal deviations to changes in the perceived timing of stimuli. Conversely, we find that in a regularly timed environment stimuli presented earlier and later than expected are perceptually shifted in a way consistent with temporal regularization. Entrainment models and models of dynamic attending have been used to capture several experimental findings with stimuli similar to the ones used here [13,34,68,69]. Dynamic attending can account for a generalized perceptual acceleration of stimuli presented at the expected time [70] due to the rhythmic deployment of attention at isochronous time points. Such an interpretation is consistent with the presence of prior-entry for attended stimuli, which leads to accelerated sensory processing [71,72]. However, an explanation based on a symmetric attentional impulse cannot account for the results reported in Experiment 2, as stimuli presented at the same anisochrony before and after the expected time point should be affected by the same acceleration.

### Monitoring Temporal Statistics

Temporal regularity has important ecological functions, i.e., in sensorimotor synchronization [10,31,73–75], musical performance [13,76,77], discrimination [78,79], and causal learning [80]. Thus it is not surprising that the brain monitors regularity across stimuli and uses the regularity of the context to modify perception as we present in this study. Exposure to regularities results in the expansion of visual working memory [81,82], improved object labelling [83], and object classification [84]. The phenomenon we report in this paper is similar to long-term adaptation effects where the context of the experiment influences our perception–and as such, perception is not absolute but relative to some internal reference [85]. Such an effect has been shown in other temporal contexts, i.e., perceived duration [50] and simultaneity [86]. Human observers have been shown to use the statistics of the environment to adjust their perception–similar to the phenomena we describe in this paper. Adaptation level theory [87,88] has been proposed to account for such instances, as the computational problem can be phrased in terms of a null point of a phenomenological dimension. When a physical stimulus corresponds to the null point, a ‘neutral’ perception is evoked. The null point, however, is subject to adaptation. Applied to our experiments, the null point should move toward being more irregular when in an irregular environment, which should have resulted in a bias to report more regular responses. This is in contradiction to the results of Experiment 1, as we find the reverse–participants reported more irregular responses in an irregular environment.

The temporal regularisation effect that we report here is consistent with a similar tendency found in the literature about perceived duration [44,50,89,90]. When asked to reproduce intervals, participants do not only employ the information provided by the current interval, but they consider the distribution of intervals they have previously been exposed to, making their response similar to the mean duration [50]. A similar phenomenon is also found in perceived simultaneity, where participants exposed to an environment comprising of biased distribution of tactile asynchronies respond to temporal order in the direction of the most frequently presented asynchrony [86]. Such tendencies to assimilate the context in the judgments is in competition with contrasting effects occurring at a ‘local’ level, where just the previous trial can influence the temporal perception of the next [91]. It has been argued that the long-term assimilation and short-term contrast effects interact with or precede one another [92,93]. According to such accounts [92], the perceptual system encodes both the prior probabilities of the stimulus distribution over time whilst also representing the recent history and redistributing resources in order to efficiently code incoming sensory signals [94,95]. Such expectation biases perception in a way that resolves any perceptual conflict with current sensory information.

### Re-focusing Time Perception

The Bayesian model of perceived timing we present in this paper advances a re-focusing of investigation towards event-based perception in the temporal domain. Historical and seminal accounts of time perception are focused on modelling time from the perspective of duration [35,37,38,44]. The model we present here is not in competition with such accounts of duration perception [50,51], but it considers a dimension that has been overlooked: the perceived timing of stimuli. We reason that expectations about the absolute timing of stimuli need to be continuously updated during a sequence, and thus they must have a fast time-course. On the other hand, long-term priors that enable expectations are interval-based and are updated after each stimulus presentation. For this reason, they have a slow time-course that can represent the statistics of the environment. The combination of the two types of information (fast and slow time-course) with sensory information improves efficiency in producing sensory estimates about temporal properties of the environment [30,96–99].

### Conclusion

In this study, we establish that the temporal regularity of the environment drives time perception. Sequences embedded in regular and irregular environments perceptually change, that is: (1) perceived regularity decreases in an irregular environment, and (2) the perceived timing of slightly irregular stimuli is modified to appear more regular if embedded in a regular environment. Using a Bayesian framework, we propose that temporal expectations about timing of stimuli are generated based on the statistics of the environment. Such expectations bias the perceived timing of stimuli in a way that results in temporal regularisation and thus a more convincing percept of having experienced a regular sequence.

## Supporting Information

S1 Table Analysis of the ‘regular’ responses as a function of jitter separately for each anisochrony and group of Experiment 1. *F* values and Bonferroni-corrected *p* values (*p* values are multiplied by 15) for one-way r.m. ANOVA on the proportion of responses in the four conditions at each level of anisochrony. Asterisks denote significant *p* values at 5% alpha level.
(PDF)

S2 Table Analysis of the ‘regular’ responses in the 0 ms jitter at each anisochrony comparing the regular and irregular groups of Experiment 1. *T* values and Bonferroni-corrected *p* values (values are multiplied by 15) for independent-sample t-tests on the proportion of responses between the two groups at each level of anisochrony. Asterisks denote significant *p* values at 5% alpha level.
(PDF)

S3 Table Descriptive statistics for the parameters obtained by fitting Yarrow et al.’s (2011) ‘two noisy criteria simultaneity model’ to the data of Experiment 1. (PDF)

## References

[1] Horr NK, Di Luca M. Filling the blanks in temporal intervals: the type of filling influences perceived duration and discrimination performance. Front Psychol. 2015;6: 114–114. 10.3389/fpsyg.2015.00114 25717310 PMC4324064

[2] Wearden JH, Norton R, Martin S, Montford-Bebb O. Internal clock processes and the filled-duration illusion. J Exp Psychol Human. 2007;33: 716–729. 10.1037/0096-1523.33.3.716 17563232

[3] Thomas EC, Brown I. Time perception and the filled-duration illusion. Percept Psychophys. Springer; 1974;16: 449–458.

[4] Schiffman HR, Bobko DJ. The role of number and familiarity of stimuli in the perception of brief temporal intervals. Am J Psychol. JSTOR; 1977;: 85–93. 871180

[5] Wearden JH, Todd NPM, Jones LA. When do auditory/visual differences in duration judgements occur? The Quarterly Journal of Experimental Psychology. 2006;59: 1709–1724. 10.1080/17470210500314729 16945856

[6] Wearden JH, Edwards H, Fakhri M, Percival A. Why “sounds are judged longer than lights”: application of a model of the internal clock in humans. Q J Exp Psychol B. 1998;51: 97–120. 10.1080/713932672 9621837

[7] Goldstone S, Lhamon WT. Studies of auditory-visual differences in human time judgment. 1. Sounds are judged longer than lights. Percept Mot Skills. 1974;39: 63–82. 10.2466/pms.1974.39.1.63 4415924

[8] Dyjas O, Ulrich R. Effects of stimulus order on discrimination processes in comparative and equality judgements: Data and models. The Quarterly Journal of Experimental Psychology. 2013. 10.1080/17470218.2013.847968 24295428

[9] Kösem A, van Wassenhove V. Temporal Structure in Audiovisual Sensory Selection. Ernst MO, editor. PLoS ONE. 2012;7: e40936 22829899 10.1371/journal.pone.0040936 PMC3400621

[10] Su Y-HY, Pöppel EE. Body movement enhances the extraction of temporal structures in auditory sequences. Psychol Res. 2012;76: 373–382. 10.1007/s00426-011-0346-3 21695472

[11] Povel DJ. Internal representation of simple temporal patterns. J Exp Psychol Human. 1981;7: 3–18. 10.1037//0096-1523.7.1.3 6452500

[12] Povel DJ. A theoretical framework for rhythm perception. Psychol Res. 1984;45: 315–337. 6728975 10.1007/BF00309709

[13] Large EW, Palmer C. Perceiving temporal regularity in music. Cogn Sci. 2002;26: 1–37. 10.1207/s15516709cog2601_1

[14] Grahn JA, Rowe JB. Finding and Feeling the Musical Beat: Striatal Dissociations between Detection and Prediction of Regularity. Cereb Cortex. 2013;23: 913–921. 10.1093/cercor/bhs083 22499797 PMC3593578

[15] Hannon EEE, Trehub SES. Tuning in to musical rhythms: infants learn more readily than adults. Proc Natl Acad Sci USA. 2005;102: 12639–12643. 10.1073/pnas.0504254102 16105946 PMC1194930

[16] Winkler I, Haden GP, Ladinig O, Sziller I, Honing H. Newborn infants detect the beat in music. Proc Natl Acad Sci USA. 2009;106: 2468–2471. 10.1073/pnas.0809035106 19171894 PMC2631079

[17] Bateson M, Kacelnik A. Starlings' preferences for predictable and unpredictable delays to food. Anim Behav. 1997;53: 1129–1142. 10.1006/anbe.1996.0388 9236010

[18] Henderson J, Hurly TA, Bateson M, Healy SD. Timing in Free-Living Rufous Hummingbirds, Selasphorus rufus. Curr Biol. 2006;16: 512–515. 10.1016/j.cub.2006.01.054 16527747

[19] Buhusi CV, Sasaki A, Meck WH. Temporal integration as a function of signal and gap intensity in rats (Rattus norvegicus) and pigeons (Columba livia). J Comp Psychol. 2002;116: 381–390. 10.1037//0735-7036.116.4.381 12539934

[20] Gallistel CR, King A, McDonald R. Sources of variability and systematic error in mouse timing behavior. J Exp Psychol Anim Behav Process. 2004;30: 3–16. 10.1037/0097-7403.30.1.3 14709111

[21] Gribova A, Donchin O, Bergman H, Vaadia E, de Oliveira SC. Timing of bimanual movements in human and non-human primates in relation to neuronal activity in primary motor cortex and supplementary motor area. Exp Brain Res. 2002;146: 322–335. 10.1007/s00221-002-1174-x 12232689

[22] Janssen P, Shadlen MN. A representation of the hazard rate of elapsed time in macaque area LIP. Nat Neurosci. 2005;8: 234–241. 10.1038/nn1386 15657597

[23] Brochard R, Tassin M, Zagar D. Got rhythm…for better and for worse. Cross-modal effects of auditory rhythm on visual word recognition. Cognition. 2013;127: 214–219. 10.1016/j.cognition.2013.01.007 23454794

[24] Correa AA, Lupiáñez JJ, Tudela PP. Attentional preparation based on temporal expectancy modulates processing at the perceptual level. Psychon Bull Rev. 2005;12: 328–334. 16082814 10.3758/bf03196380

[25] Doherty JR, Rao A, Mesulam MM, Nobre AC. Synergistic effect of combined temporal and spatial expectations on visual attention. J Neurosci. 2005;25: 8259–8266. 10.1523/JNEUROSCI.1821-05.2005 16148233 PMC6725546

[26] Escoffier N, Sheng DYJ, Schirmer A. Unattended musical beats enhance visual processing. Acta Psychol. Elsevier B.V; 2010;135: 12–16. 10.1016/j.actpsy.2010.04.005 20451167

[27] Grill-Spector K, Henson R, Martin A. Repetition and the brain: neural models of stimulus-specific effects. Trends Cogn Sci. 2006;10: 14–23. 10.1016/j.tics.2005.11.006 16321563

[28] Cravo AM, Rohenkohl G, Wyart V, Nobre AC. Temporal Expectation Enhances Contrast Sensitivity by Phase Entrainment of Low-Frequency Oscillations in Visual Cortex. J Neurosci. 2013;33: 4002–4010. 10.1523/JNEUROSCI.4675-12.2013 23447609 PMC3638366

[29] Rohenkohl G, Nobre AC. Alpha Oscillations Related to Anticipatory Attention Follow Temporal Expectations. J Neurosci. 2011;31: 14076–14084. 10.1523/JNEUROSCI.3387-11.2011 21976492 PMC4235253

[30] VanRullen R, Dubois J. The psychophysics of brain rhythms. Front Psychol. 2011;2: 1–10. 10.3389/fpsyg.2011.00203 21904532 PMC3163286

[31] Repp BHB. Sensorimotor synchronization: a review of the tapping literature. Psychon Bull Rev. 2005;12: 969–992. 16615317 10.3758/bf03206433

[32] McNeill WH. Keeping together in time: Dance and drill in human history. Cambridge, MA: Harvard Univ. Press; 1995.

[33] Karmarkar UR, Buonomano DV. Timing in the Absence of Clocks: Encoding Time in Neural Network States. Neuron. 2007;53: 427–438. 10.1016/j.neuron.2007.01.006 17270738 PMC1857310

[34] Buonomano DV, Merzenich MM. Temporal information transformed into a spatial code by a neural network with realistic properties. Science. 1995;267: 1028–1030. 7863330 10.1126/science.7863330

[35] Gibbon J, Church RM, Meck WH. Scalar timing in memory. Ann NY Acad Sci. Wiley Online Library; 1984;423: 52–77. 10.1111/j.1749-6632.1984.tb23417.x 6588812

[36] Gibbon J. Scalar expectancy theory and Weber's law in animal timing. Psychol Rev. American Psychological Association; 1977;84: 279.

[37] Creelman CD. Human discrimination of auditory duration. J Acoust Soc Am. 1962;34: 582.

[38] Treisman M. Temporal discrimination and the indifference interval. Implications for a model of the "internal clock". Psychol Monogr. 1963;77: 1–31. 10.1037/h0093864 5877542

[39] Wackermann J, Ehm W. The dual klepsydra model of internal time representation and time reproduction. J Theor Biol. 2006;239: 482–493. 10.1016/j.jtbi.2005.08.024 16202427

[40] van Rijn H, Gu B-M, Meck WH. Dedicated Clock/Timing-Circuit Theories of Time Perception and Timed Performance Advances in Experimental Medicine and Biology. New York, NY: Springer New York; 2014 pp. 75–99. 10.1007/978-1-4939-1782-2_5 25358706

[41] Meck WH. Neuropsychology of timing and time perception. Brain Cogn. 2005;58: 1–8. 10.1016/j.bandc.2004.09.004 15878722

[42] Teki S, Grube M, Griffiths TD. A unified model of time perception accounts for duration-based and beat-based timing mechanisms. Front Integr Neurosci. Frontiers Media SA; 2011;5. 10.3389/fnint.2011.00090 PMC3249611 22319477

[43] Buhusi CV, Meck WH. What makes us tick? Functional and neural mechanisms of interval timing. Nat Rev Neurosci. 2005;6: 755–765. 10.1038/nrn1764 16163383

[44] Shi Z, Church RM, Meck WH. Bayesian optimization of time perception. Trends Cogn Sci. Elsevier Ltd; 2013;17: 556–564. 10.1016/j.tics.2013.09.009 24139486

[45] Knill DC, Richards W. Perception as Bayesian Inference. Cambridge, UK: Cambridge University Press; 1996.

[46] Mamassian P, Landy MS, Maloney LT. Bayesian modelling of visual perception Probabilistic models of the brain: Perception and neural function. Cambridge, MA: MIT Press; 2002 pp. 13–36.

[47] Wolpert DM, Ghahramani Z. Computational principles of movement neuroscience. Nat Neurosci. 2000;3 Suppl: 1212–1217. 10.1038/81497 11127840

[48] Kersten D, Yuille A. Bayesian models of object perception. Curr Opin Neurobiol. 2003;13: 150–158. 10.1016/S0959-4388(03)00042-4 12744967

[49] Petzschner FH, Glasauer S. Iterative Bayesian Estimation as an Explanation for Range and Regression Effects: A Study on Human Path Integration. J Neurosci. 2011;31: 17220–17229. 10.1523/JNEUROSCI.2028-11.2011 22114288 PMC6623840

[50] Jazayeri M, Shadlen MN. Temporal context calibrates interval timing. Nat Neurosci. Nature Publishing Group; 2010;13: 1020–1026. 10.1038/nn.2590 PMC2916084 20581842

[51] Petzschner FH, Glasauer S, Stephan KE. A Bayesian perspective on magnitude estimation. Trends Cogn Sci. Elsevier Ltd; 2015;19: 285–293. 10.1016/j.tics.2015.03.002 25843543

[52] Shi Z, Burr D. Predictive coding of multisensory timing. Current Opinion in Behavioral Sciences. Elsevier Ltd; 2016;: 1–7. 10.1016/j.cobeha.2016.02.014 PMC5040498 27695705

[53] Freestone DM, Church RM. Optimal timing. Current Opinion in Behavioral Sciences. Elsevier Ltd; 2016;: 1–6. 10.1016/j.cobeha.2016.02.031

[54] Cicchini GM, Arrighi R, Cecchetti L, Giusti M, Burr DC. Optimal Encoding of Interval Timing in Expert Percussionists. J Neurosci. 2012;32: 1056–1060. 10.1523/JNEUROSCI.3411-11.2012 22262903 PMC6621155

[55] Acerbi L, Wolpert DM, Vijayakumar S. Internal Representations of Temporal Statistics and Feedback Calibrate Motor-Sensory Interval Timing. Maloney LT, editor. PLoS Comput Biol. 2012;8: e1002771 10.1371/journal.pcbi.1002771.s003 23209386 PMC3510049

[56] Jazayeri M, Shadlen MN. A Neural Mechanism for Sensing and Reproducing a Time Interval. Curr Biol. Elsevier Ltd; 2015;: 1–12. 10.1016/j.cub.2015.08.038 PMC4618078 26455307

[57] Schulze HH. The perception of temporal deviations in isochronic patterns. Percept Psychophys. 1989;45: 291–296. 2710629 10.3758/bf03204943

[58] Hoopen ten GG, Hartsuiker RR, Sasaki TT, Nakajima YY, Tanaka MM, Tsumura TT. Auditory isochrony: time shrinking and temporal patterns. Perception. 1995;24: 577–593. 7567431 10.1068/p240577

[59] Hoopen Ten G, Van Den Berg S, Memelink J, Bocanegra B, Boon R. Multiple-look effects on temporal discrimination within sound sequences. Atten Percept Psychophys. 2011;73: 2249–2269. 10.3758/s13414-011-0171-1 21735312 PMC3204043

[60] Yarrow K, Jahn N, Durant S, Arnold DH. Shifts of criteria or neural timing? The assumptions underlying timing perception studies. Conscious Cogn. Elsevier Inc; 2011;20: 1518–1531. 10.1016/j.concog.2011.07.003 21807537

[61] Pelli DG. The VideoToolbox software for visual psychophysics: transforming numbers into movies. Spat Vis. 1997;10: 437–442. 9176953

[62] Brainard DH. The psychophysics toolbox. Spat Vis. 1997;10: 433–436. 9176952

[63] Miller JJ, Ulrich RR. On the analysis of psychometric functions: the Spearman-Kärber method. Percept Psychophys. 2001;63: 1399–1420. 11800465 10.3758/bf03194551

[64] Dienes Z. Using Bayes to get the most out of non-significant results. Front Psychol. 2014. 10.3389/fpsyg.2014.00781 PMC4114196 25120503

[65] Rouder JN, Speckman PL, Sun D, Morey RD, Iverson G. Bayesian t tests for accepting and rejecting the null hypothesis. Psychon Bull Rev. 2009;16: 225–237. 10.3758/PBR.16.2.225 19293088

[66] Sternberg S, Knoll RL. The perception of temporal order: Fundamental issues and a general model. Attention and performance IV. 1973;: 629–685.

[67] Baddeley A. Working memory: The interface between memory and cognition. J Cogn Neurosci. MIT Press; 1992;4: 281–288. 10.1162/jocn.1992.4.3.281 23964884

[68] Miall C. The storage of time intervals using oscillating neurons. Neural Comput. MIT Press; 1989;1: 359–371.

[69] Jones MR, Boltz M. Dynamic Attending and Responses to Time. Psychol Rev. 1989;96: 459–491. 2756068 10.1037/0033-295x.96.3.459

[70] Li MS, Rhodes D, Di Luca M. For the Last Time: Temporal Sensitivity and Perceived Timing of the Final Stimulus in an Isochronous Sequence. Timing Time Percept. 2016 10.1163/22134468-00002057

[71] Spence C, Parise C. Prior-entry: A review. Conscious Cogn. Elsevier Inc; 2010;19: 364–379. 10.1016/j.concog.2009.12.001 20056554

[72] Sternberg S, Knoll RL, Gates BA. Prior entry reexamined: Effect of attentional bias on order perception. 1971.

[73] Merker BH, Madison GS, Eckerdal P. On the role and origin of isochrony in human rhythmic entrainment. Cortex. Elsevier; 2009;45: 4–17. 10.1016/j.cortex.2008.06.011 19046745

[74] Manning F, Schutz M. “Moving to the beat” improves timing perception. Psychon Bull Rev. 2013 10.3758/s13423-013-0439-7 23670284

[75] Elliott MT, Wing AM, Welchman AE. Moving in time: Bayesian causal inference explains movement coordination to auditory beats. Proc R Soc Lond B Biol Sci. 2014;281: 20140751–20140751. 10.1098/rspb.2014.0751 PMC4046422 24850915

[76] Honing H. Structure and Interpretation of Rhythm in Music In: Deutsch D, editor. 3rd ed London, UK: Academic Press; 2013 pp. 369–404. 10.1016/B978-0-12-381460-9.00009-2

[77] Merchant H, Grahn J, Trainor L, Rohrmeier M, Fitch WT. Finding the beat: a neural perspective across humans and non-human primates. Phil Trans R Soc Lond B Biol Sci. 2015;370: 20140093–20140093. 25646516 10.1098/rstb.2014.0093 PMC4321134

[78] Kusnir F, Chica AB, Mitsumasu MA, Bartolomeo P. Consciousness and Cognition. Conscious Cogn. Elsevier Inc; 2011;20: 1201–1210. 10.1016/j.concog.2011.01.012 21349743

[79] Rohenkohl G, Gould IC, Pessoa J, Nobre AC. Combining spatial and temporal expectations to improve visual perception. J Vis. 2014;14: 8–8. 10.1167/14.4.8 PMC3983934 24722562

[80] Greville WJ, Buehner MJ. Temporal predictability facilitates causal learning. J Exp Psychol Gen. 2010;139: 756–771. 10.1037/a0020976 21038987

[81] Brady TF, Konkle T, Alvarez GA. Compression in visual working memory: Using statistical regularities to form more efficient memory representations. J Exp Psychol Gen. 2009;138: 487–502. 10.1037/a0016797 19883132

[82] Umemoto A, Scolari M, Vogel EK, Awh E. Statistical learning induces discrete shifts in the allocation of working memory resources. J Exp Psychol Human. 2010;36: 1419–1429. 10.1037/a0019324 PMC2990794 20718564

[83] Estes KG, Evans JL, Alibali MW, Saffran JR. Can infants map meaning to newly segmented words? Statistical segmentation and word learning. Psychol Sci. 2007;18: 254–260. 17444923 10.1111/j.1467-9280.2007.01885.x PMC3864753

[84] Turk-Browne NB, Scholl BJ, Johnson MK, Chun MM. Implicit Perceptual Anticipation Triggered by Statistical Learning. J Neurosci. 2010;30: 11177–11187. 10.1523/JNEUROSCI.0858-10.2010 20720125 PMC2947492

[85] Dyjas O, Ulrich R. Effects of stimulus order on discrimination processes in comparative and equality judgements: Data and models. The Quarterly Journal of Experimental Psychology. 2014;67: 1121–1150. 10.1080/17470218.2013.847968 24295428

[86] Miyazaki M, Yamamoto S, Uchida S, Kitazawa S. Bayesian calibration of simultaneity in tactile temporal order judgment. Nat Neurosci. 2006;9: 875–877. 10.1038/nn1712 16732276

[87] Helson H. Adaptation-level as frame of reference for prediction of psychophysical data. Am J Psychol. 1947;60: 1–29. 20288861

[88] Helson H. Adaptation-level theory: an experimental and systematic approach to behavior. New York: Harper & Row; 1964.

[89] Hartcher-O'Brien J, Di Luca M, Ernst MO. The duration of uncertain times: audiovisual information about intervals is integrated in a statistically optimal fashion. PLoS ONE. 2014;9: e89339 10.1371/journal.pone.0089339 24594578 PMC3942383

[90] Miyazaki M, Nozaki D, Nakajima Y. Testing Bayesian models of human coincidence timing. J Neurophysiol. 2005;94: 395–399. 15716368 10.1152/jn.01168.2004

[91] Van der Burg E, Alais D, Cass J. Rapid Recalibration to Audiovisual Asynchrony. J Neurosci. 2013;33: 14633–14637. 10.1523/JNEUROSCI.1182-13.2013 24027264 PMC6705173

[92] Chopin A, Mamassian P. Predictive Properties of Visual Adaptation. Curr Biol. Elsevier Ltd; 2012;22: 622–626. 10.1016/j.cub.2012.02.021 22386314

[93] Yamamoto S, Miyazaki M, Iwano T, Kitazawa S. Bayesian Calibration of Simultaneity in Audiovisual Temporal Order Judgments. Ernst MO, editor. PLoS ONE. 2012;7: e40379 10.1371/journal.pone.0040379.g005 22792297 PMC3392227

[94] Barlow HB. The coding of sensory messages In: Thorpe WH, Zangwill OL, editors. Current problems in animal behaviour. Cambridge: Cambridge University Press; 1961 pp. 330–360.

[95] Wainwright MJ. Visual adaptation as optimal information transmission. Vision Res. 1999;39: 3960–3974. 10748928 10.1016/s0042-6989(99)00101-7

[96] Wei XX, Stocker A. Efficient coding provides a direct link between prior and likelihood in perceptual Bayesian inference. In: Bartlett PL, Pereira F, Burges C, Botou L, Weinberger KQ, editors. 2012 pp. 1313–1321.

[97] Wei X-X, Stocker AA. A Bayesian observer model constrained by efficient coding can explain “anti-Bayesian” percepts. Nat Neurosci. Nature Publishing Group; 2015;: 1–11. 10.1038/nn.4105 26343249

[98] Friston K. A theory of cortical responses. Phil Trans R Soc Lond B Biol Sci. 2005;360: 815–836. 15937014 10.1098/rstb.2005.1622 PMC1569488

[99] Friston K. The free-energy principle: a unified brain theory? Nat Rev Neurosci. Nature Publishing Group; 2010;11: 127–138. 10.1038/nrn2787 20068583

