# Correction: Temporal Regularity of the Environment Drives Time Perception

**DOI:** 10.1371/journal.pone.0161677

**Published:** 2016-08-17

**Authors:** Darren Rhodes, Massimiliano Di Luca

The Data Availability statement for this paper is incorrect. The correct statement is: All relevant data for the experiments described in the paper are available for download both at https://osf.io/zy6uc/ and at co-author DR’s personal website (http://www.darrenrhodes.org/open-data.html). The publisher apologizes for the error.
