# Supplementary material for: Temporal Regularity of the Environment Drives Time Perception
Source: PLoS One. 2016 Jul 21;11(7):e0159842. doi: 10.1371/journal.pone.0159842 (PMC4956244; doi:10.1371/journal.pone.0159842)
Supplement: S1 Table — F values and Bonferroni-corrected p values (p values are multiplied by 15) for one-way r.m. ANOVA on the proportion of responses in the four conditions at each level of anisochrony. Asterisks denote significant p values at 5% alpha level. (PDF) [file pone.0159842.s001.pdf]

Supporting Information S1

S1 Table. Analysis of the 'regular' responses as a function of jitter separately for each anisochrony and group of Experiment 1. *F* values and Bonferroni-corrected *p* values (*p* values are multiplied by 15) for one-way r.m. ANOVA on the proportion of responses in the four conditions at each level of anisochrony. Asterisks denote significant *p* values at 5% alpha level.

Anisochrony [ms]	Regular group			Irregular group		
	$F_{3,27}=$	$p=$	$\eta_p^2=$	$F_{3,27}=$	$p=$	$\eta_p^2=$
-200	0.7	>0.99	0.07	4.2	0.228	0.32
-150	0.3	>0.99	0.04	0.7	>0.99	0.07
-100	0.1	>0.99	0.02	7.1	*0.017	0.44
-80	1.2	>0.99	0.12	11.9	*0.001	0.57
-60	12.3	*0.001	0.58	25.5	*0.001	0.74
-40	4.3	0.198	0.32	37.0	*0.001	0.80
-20	14.0	*0.001	0.61	25.2	*0.001	0.74
0	7.1	*0.017	0.44	24.4	*0.001	0.73
20	6.1	*0.039	0.40	32.7	*0.001	0.78
40	12.1	*0.001	0.57	26.0	*0.001	0.74
60	6.7	*0.023	0.43	38.8	*0.001	0.81
80	0.1	>0.99	0.01	12.8	*0.001	0.59
100	1.4	>0.99	0.14	9.4	*0.003	0.51
150	0.2	>0.99	0.02	4.3	0.208	0.32
200	3.4	0.473	0.28	0.2	>.99	0.02
