# Supplementary material for: Temporal Regularity of the Environment Drives Time Perception
Source: PLoS One. 2016 Jul 21;11(7):e0159842. doi: 10.1371/journal.pone.0159842 (PMC4956244; doi:10.1371/journal.pone.0159842)
Supplement: S2 Table — T values and Bonferroni-corrected p values (values are multiplied by 15) for independent-sample t-tests on the proportion of responses between the two groups at each level of anisochrony. Asterisks denote significant p values at 5% alpha level. (PDF) [file pone.0159842.s002.pdf]

Supporting Information S2

S2 Table. Analysis of the "regular" responses in the 0ms jitter at each anisochrony comparing the regular and irregular groups of Experiment 1. *T* values and Bonferroni-corrected *p* values (values are multiplied by 15) for independent-sample t-tests on the proportion of responses between the two groups at each level of anisochrony. Asterisks denote significant *p* values at 5% alpha level.

Anisochrony [ms]	0ms jitter – Difference between groups	
	T_{18} =	p=
-200	-0.6	0.556
-150	-0.1	0.237
-100	-0.3	0.760
-80	-0.3	0.778
-60	-0.4	0.671
-40	-2.6	*0.019
-20	-3.9	*0.001
0	-2.1	*0.047
20	-2.5	*0.021
40	-1.2	0.242
60	-0.6	0.546
80	-0.1	0.922
100	-1.0	0.336
150	-0.4	0.680
200	0.5	0.567
