# Supplementary material for: Temporal Regularity of the Environment Drives Time Perception
Source: PLoS One. 2016 Jul 21;11(7):e0159842. doi: 10.1371/journal.pone.0159842 (PMC4956244; doi:10.1371/journal.pone.0159842)
Supplement: S3 Table — (PDF) [file pone.0159842.s003.pdf]

Supporting Information S3

S3 Table. Descriptive statistics for the parameters obtained by fitting Yarrow et al.'s (2011) 'two noisy criteria simultaneity model' to the data of Experiment 1.

Parameter	Mean		Standard Error of the Mean	
	Regular	*Irregular*	*Regular*	*Irregular*
Threshold Early	-80.1	-83.8	14.2	8.9
Threshold Late	83.1	95.2	12.9	12.5
Slope Early	58.9	38.2	10.8	5.1
Slope Late	74.5	43.7	12.4	4.4
